# PPI in LifeMap-QUEST: an example of co-producing videos in different languages to support inclusion in a clinical study

**DOI:** 10.1186/s40900-026-00858-9

**Published:** 2026-03-11

**Authors:** David C. Clayton, William B. Nicolson, Sarah Anthony, Rachel Hobson, LifeMap-QUEST PPI Advisory Group, David Batchelor, Khandu Chauhan, Sharon Hack, Tony Locke, Noorie Majothi, Harish Mistry, Kirit Mistry, Pratiba Mkdami, Nina Prajapati, Harold Palmer, Ballu Patel, Keval Sachdev, Purshottam Vallabh, Ranjan Vallabh, G. André Ng

**Affiliations:** 1 Cardiovascular Sciences, School of Medical Sciences, University of Leicester, University Road, Leicester, LE1 7RH UK; 2 Cardiology, University Hospitals of Leicester NHS Trust, University of Leicester, University Road, Leicester, LE1 7RH UK; 3 NIHR Leicester Biomedical Research Centre, BHF Leicester Centre of Research Excellence, University of Leicester, University Road, Leicester, LE1 7RH UK

**Keywords:** Clinical trials, Ethnic minorities, PPI, Inclusion, Co-production, Translation, Participant information, Videos

## Abstract

Recruiting participants into clinical trials is often challenging, particularly when trying to involve people from diverse or disadvantaged communities. This article describes how we co‑produced video resources to support inclusion in the LifeMap‑QUEST clinical study. LifeMap is a new approach to diagnosing people who may be at risk of Sudden Cardiac Death (SCD). Recognising the longstanding under‑representation of ethnic minority groups in research, and the barriers posed by language, literacy, and trust, the LifeMap‑QUEST Patient and Public Involvement (PPI) Advisory Group worked with community partners and clinicians to develop participant information videos in English, Gujarati, and Hindi. This article explains how this collaborative process unfolded. As part of our co‑production approach, we tested AI translation tools such as ChatGPT. However, the translations produced were too formal, used complex terminology, and did not capture the tone or cultural context needed. We therefore adopted a collaborative method with a local charity, South Asian Health Action (SAHA), using forward–back translation, validation by native speakers within the PPI Advisory Group, and review by cardiac consultants who spoke Gujarati and Hindi. This ensured both accuracy and cultural appropriateness. Filming used a simple “talking‑head” style to create a friendly, approachable tone. Members of the PPI Advisory Group reviewed the videos and offered constructive feedback. The article highlights the challenges of developing participant information in multiple languages, emphasising the need for PPI, community partners, and expert clinical oversight. We hope that by describing what we did and the lessons we learnt, it will support others to make participant information videos in different languages.

**Supplementary Information:**

The online version contains supplementary material available at 10.1186/s40900-026-00858-9.

## Abstract (plain language summary)

This article explains how our Patient and Public Involvement (PPI) Advisory Group worked with researchers to create study information videos in English, Gujarati, and Hindi for a project called LifeMap‑QUEST. Written leaflets inviting people to take part in research can be long, confusing, and difficult to understand. This can be especially difficult for people who do not speak or read English well. In Leicester, where we are carrying out the study, many people speak a South Asian language better than English. To encourage South Asian people to take part in our study, our PPI Advisory Group suggested creating short videos in different languages. This article describes the journey of how we worked together, what we did, and why it matters. As a first step, we used modern technology called ChatGPT to turn our information into other languages but the language it produced was too technical and not easy for people to follow. Instead, we worked closely with our PPI Advisory Group, a community charity called South Asian Health Action (SAHA), and cardiologists who speak Gujarati and Hindi. Together, we checked the translations carefully to make sure the words were accurate, clear, and friendly. The videos were filmed in a warm and approachable style, with people speaking directly to the camera. We asked our PPI Advisory Group to review the videos, and they told us we needed clearer sound and better images to help viewers follow what was being explained. Their feedback helped us improve the final versions. We hope that by describing what we did and the lessons we learnt, it will support others to make videos in different languages.

**Supplementary Information:**

The online version contains supplementary material available at 10.1186/s40900-026-00858-9.

## Introduction

Clinical trials drive innovation in healthcare, underpinning advances in the prevention, diagnosis, and treatment of medical conditions. It is therefore scientifically and ethically important to recruit a diverse range of people to them [1]. Without diversity, concerns exist scientifically on the generalisability of findings from clinical trials, their statistical power and whether they are biased because they are not truly representative of the population [1–3]. Important cultural issues or health problems relevant to the care of ethnic minority groups, for example, may be missed without their representation in clinical trials [4]. To address this, health researchers need strategies to encourage the participation of ethnic minority groups in clinical trials.

This article describes an approach taken to co-produce videos to support the recruitment of people from the South Asian Community to a clinical study called LifeMap-QUEST^1^. LifeMap is a diagnostic system which has been developed to assess the risk of Sudden Cardiac Death (SCD) [5–7]. Working collaboratively with our Patient and Public Involvement (PPI) Advisory Group, a community partner, and clinicians, three ‘talking head’ videos in English, Gujarati and Hindi were created to provide participants with patient information about the LifeMap-QUEST study to encourage participation.

## Background

A key aspect of the LifeMap-QUEST study was ensuring diversity within our PPI Advisory Group. The group consists of 14 members living with heart conditions, 10 of whom are from the South Asian community. The membership includes nine men and five women, representing a broad age range spanning both working-age adults and those over retirement age.

The PPI Advisory Group, including a PPI co-applicant, was established locally in Leicester City with support from the NIHR Biomedical Research Centre (BRC), the NIHR Research Support Service (RSS), and a community charity called SAHA^2^. As new PPI contributors joined, they also drew on their own networks and social media platforms to help ‘snowball’ interest and encourage wider participation in the study. (See poster presented at ICTMC 2024, Additional File 1)

Coordination and planning of PPI activities was undertaken by a PPI co-application and a full-time PPI Research Associate. The PPI Advisory group played a central role in shaping the design of the wearable device—the LifeMap-Vest—used as part of the LifeMap diagnostic system. The clinical trial evaluating the LifeMap-Vest will take place across four sites in England, including Leicester, a city with a large South Asian population.

Our PPI Advisory Group emphasised that English is not the first language for many South Asian residents of Leicester, particularly recent migrants and older adults. They advised that providing study information through video, delivered in a range of South Asian languages, would help make the research more accessible and encourage participation. Leicester is a linguistically diverse city where more than 200 languages are spoken, including many South Asian languages; Gujarati is the most widely spoken Asian language in the city [8].

Existing evidence shows that people from South Asian communities have a higher risk of coronary heart disease [9–10]. Research also indicates that rates of ICD implantation, an important treatment to prevent SCD, are lower in South Asian groups compared with white populations [11]. Ensuring participation from this community in the LifeMap-QUEST study was therefore considered important for the study.

SAHA, as a local community organisation and charity, had previously produced health-related videos in a range of South Asian languages. This experience created a valuable opportunity to collaborate with them on developing multilingual participant information videos for LifeMap-QUEST. The LifeMap-QUEST research team were asked to provide support for video production. Despite limited resources, they agreed to allocate resources to make three videos, one in English and two in South Asian languages, for use at the Leicester study site.

The research team also felt that involving cardiac consultants from the hospital who spoke the relevant languages was important. Their involvement could help ensure clinical accuracy and provide reassurance to confirm the quality of the translated content. Filming was carried out with support from colleagues at the University of Leicester’s Institute for Precision Health^3^ to provide technical and quality assurance.

Along with an English video, the Asian languages included and agreed for the videos were Gujarati (the main Asian Language spoken in Leicester) and Hindi (an Asian Language thought to ‘cross over’ to some other Asian Languages)^4^.

### Rationale for creating the videos

Participant information in clinical trials is typically provided in a lengthy, written format, and does not always support comprehension for people who lack literacy and language skills [12]. This may deter non-English speaking/reading people or people whose first language is not English from taking part in clinical trials [13]. Even amongst those who give consent to participate, there may be poor comprehension or misunderstanding of what they are consenting to do [14]. Offering participant information in video format and different languages, therefore, may be one solution to help increase comprehension, build trust and reduce language barriers [15].

Systematic reviews have shown that audio‑visual resources, such as videos, produce only modest improvements in participants’ knowledge and understanding within clinical trials, and the overall quality of this evidence remains low [16–19]. Nonetheless, some studies indicate that when specifically tailored for ethnic minority groups, audio‑visual interventions can enhance knowledge, generate positive attitudes, and support greater diversity in clinical trial participation [2, 20].

Trust can be a major barrier to recruiting ethnic minority groups to research [21]. A systematic review of the use of videos in cancer trials highlighted that they can help address trust concerns amongst ethnic minority groups [1]. Other studies (mostly in the US) have also found that videos or other multimedia aimed at ethnic minority groups, including those with different languages other than English, may help increase participation in research [22–23] or reduce anxiety in undertaking medical procedures [24–25]. As Skinner et al. (2019) argue, for videos to make a difference in understanding and participation with ethnic minorities in clinical trials, messages need to be ‘tailored’. They are unlikely to lead to greater participation unless they are culturally sensitive and address the issue of trust in underrepresented groups [26].

### Our co-production approach to creating the videos

Recognising the need for cultural competence in developing our videos, we sought to involve PPI contributors, a community partner, and clinicians in co‑producing the LifeMap‑QUEST participant information videos. Our approach for embedding cultural competence was informed by involvement frameworks, including the principles of PPI and co‑production. For example, the UK Health Research Authority recommends involving public contributors with relevant lived experience to ensure that participant information materials are accessible, relevant, and understandable [5]. Wider guidance on involving people in research, such as that from NIHR, emphasises the ethos of “doing with” rather than “doing to,” promoting participatory and inclusive practices [27].

The UK Standards for Public Involvement [28] further embed shared values central to PPI, highlighting the importance of drawing on diverse perspectives, providing adequate support for contributors, and ensuring that their involvement makes a meaningful difference. The standard of “working together” is particularly relevant, developing mutually respectful relationships and participatory ways of working. In parallel, NIHR’s principles of co‑production [29], including sharing power, enabling reciprocity, and fostering joint ownership of processes and outputs, shaped how we structured collaboration within the project. Co‑production sits on a spectrum of involvement and centres on collaborative partnerships between members of the public, patients, carers, communities, clinicians, and researchers [30].

Although ultimate responsibility for participant information materials remained with the research team, we intended to actively support contributors to have an equal voice in shaping the videos, ensuring their perspectives carried equal weight to those of researchers [31, 32]. Our approach was also informed by asset‑based models of co‑production [33], which harness the strengths within individuals and communities. In practice, this meant recognising and mobilising the PPI Advisory Groups and community partners’ linguistic expertise and cultural understanding to help ensure the video content was culturally competent and more likely to resonate with South Asian communities. Our participatory translation exemplified this ethos (see below).

### Aim of this article

This article aims to highlight our co‑production approach to developing the videos. It describes how we involved our PPI contributors and community partners, outlining the activities that supported meaningful co‑production. We also share lessons learned for researchers developing similar participant information resources and discuss the benefits of our approach. The article does not present a scientific evaluation of the videos or the co‑production process. Rather, it illustrates how PPI informed the translation, design, and development of the videos. No formal data were collected from contributors or partners. Instead, this work is offered as an example of good involvement practice within clinical trials research, providing insights that may be transferable to other studies and to wider PPI practice.

## Methods

There were two stages to our process of creating the videos.

1. Participatory translation of the participant information.
2. Filming, editing and evaluation of the videos to create the ‘final cut’.

### Participatory translation of the participant information

In the initial stage, a number of activities were undertaken to inform the process. Table 1 describes the PPI activities in detail. The activities involved several face-to-face meetings to capture discussions to inform what was included and how this was translated.

As a first step, we used AI technology to see if this could provide the translation and used ChatGPT to turn our patient information into another language (Gujarati). However, the language it produced was too technical and not easy for our PPI contributors to follow.

A shorter English participant information sheet was then created to act as the ‘script’ for SAHA to undertake translations. There was a consensus that the videos should not be too long (approximately four to five minutes in running time), otherwise people would be ‘put off’.

The participatory translation activity was ‘forward/back translation’[10]. This method sees a ‘forward translation’ from the main language (English) to the intended language(e.g. Hindi) and then a ‘back translation’ from the intended language (e.g. Hindi) back to the main one (English). A comparison between the two is undertaken to check for communication and translation errors.

**Table 1:** Activities for participatory translation of the participant information

Activity	Aim	Partners	Methods
Testing ChatGPT for translations	To explore whether ChatGPT could translate patient information for use in the videos	12 PPI contributors (8 with Asian-language skills incl. Gujarati); 2 research team members	Meeting and focused discussion: A page of participant information was translated into Gujarati using ChatGPT; contributors compared the translation with the English version and discussed accuracy and suitability
Creating the script for the videos	To develop a shorter English participant information script suitable for video translation	12 PPI contributors; 2 research team members	Meeting and focused discussion: Draft script reviewed in a group discussion; contributors suggested ways to shorten, simplify and make the information more accessible; amended script circulated for email comment, then finalised (See additional file 2)
PPI validation -forward/back translations	To check the accuracy and clarity of the Gujarati and Hindi translations provided by SAHA	5 contributors fluent in Gujarati/Hindi; 2 research team members; 2 representatives from SAHA	Meeting and focused discussion: Forward and back translations of the ‘script’; contributors compared English and translated audio versions, identifying issues and suggesting clearer, lay-friendly alternatives; provided to SAHA for revised translations
Involving cardiac consultants	To ensure medical accuracy and clarity of translated videos	2 cardiac consultants (Gujarati and Hindi speaking)	Email correspondence: The consultants reviewed the translated audio recordings and provided email feedback on medical terminology and phrasing. SAHA used the feedback to revise the translations

PPI Contributors from the LifeMap-QUEST PPI Advisory Group were contacted by email with an outline of the activity. Participation was self-selecting, with those PPI contributors who spoke Asian languages encouraged to join the activities where these skills were needed. Those without these language skills were still able to attend and join in the discussions. Contributors were provided with a ‘thank you’ cash payment based on the recommended NIHR rate [34].

Contributions from the cardiac consultants were recruited by a member of the research team who was a cardiac consultant. Given time constraints, email correspondence was used rather than a meeting.

### Filming, editing and evaluation of the videos to create the ‘final cut’

Once the translations were finalised, the filming and editing of the videos could begin in stage two. Table 2 outlines the activities involved in filming, editing, and evaluating the videos. During this phase, collaboration was with individuals from SAHA to ensure the videos demonstrated cultural competence and the Institute of Precision Health for filming and editing. A community location was chosen, and an agreement reached that the translators would present their scripts seated and directly to the camera. The aim was to make the videos informal and friendly and enable them to be delivered in a personable way.

Once filming and editing were completed, an evaluation activity was held with the PPI contributors. The purpose of the session was to give contributors, including those not involved in the translations, an opportunity to view the final videos and suggest improvements.

**Table 2:** Activities undertaken for filming, editing and evaluating the videos

Activity	Aim	Partners	Methods
Meeting to discuss filming	To plan the filming of the videos and agree format	4 representatives from SAHA; 1 person from the Institute of Precision Health; 1 research team member	Online meeting and focused discussion: Agreed location of filming, ‘talking head’ format, backgrounds and cultural attire
Filming and editing	To film Gujarati, Hindi and English versions of the LifeMap-QUEST videos	3 representatives from SAHA; 1 camera operator from the Institute of Precision Health; 1 research team member	Filmed “talking head” videos over one day; 1–2 takes per script. A further 3 days were required to make edits, add headings, images, and supplementary footage to enhance clarity and engagement
PPI evaluation of videos	To gather feedback on whether videos were culturally appropriate and encouraging for potential participants	13 PPI contributors; 6 research team members; 1 representative from SAHA	Meeting and focused discussion: Viewing of videos during a LifeMap-QUEST PPI workshop All contributors completed a short evaluation questionnaire while watching the videos to facilitate discussion, followed by group suggestions for improvements to be made. These were passed to the Institute of Precision Health for further edits
Final cut review	To confirm heading placement and final refinements.	2 PPI contributors; 1 research team member	Meeting and focused discussion: Contributors viewed near-final videos and advised on timings and placement of headings; passed to the Institute of Precision Health for final edits

The videos were shown on a large screen, and contributors were invited to complete a short questionnaire designed by the research team. The questionnaire acted as a prompt, asking contributors to rate (1 = poor, 5 = excellent) key aspects of the videos the research team and editors wished to assess, such as the style, sound quality, images used and translations. Contributors were also asked to select one overall rating statement:

- The video is poor and unlikely to encourage participation in LifeMap-QUEST.
- The video is acceptable but needs further editing to encourage participation.
- The video is good, and I would be interested in taking part in LifeMap-QUEST after viewing it.
- The video is excellent, highly engaging, and I would like to watch it again. (see Additional File 3 for the full version)

This activity was intended as a PPI engagement activity to facilitate discussion rather than a formal assessment of the videos’ effectiveness. By collecting these scores on the videos, the questionnaire helped direct further edits to improve the videos for use with the clinical trial, at least from the perspective of the PPI contributors. A more robust evaluation of the videos would be needed after the clinical trial to fully assess their effectiveness.

Following the video evaluation, a brief meeting with two PPI contributors (who also took part in the video workshop) provided additional advice on the final edits to ensure any images were included in the right places for the dialogue.

Once completed, the final videos were circulated to PPI contributors for any final comments and given to the research team to ‘sign off’ for use in the LifeMap-QUEST clinical study, ahead of formal ethical approval.

## Results

Our approach to co-producing the videos led to several changes in the translation and creation of the videos, driven largely by the involvement of PPI contributors and other partners. Below are some key findings, accompanied by lessons that may support other researchers producing videos translated into multiple languages.

### Don’t rely solely on digital tools for translations

When Gujarati speaking PPI contributors reviewed the initial ChatGPT translations, they identified several issues. These included incorrect or poorly interpreted medical terminology, for example, the translation of *implantable cardioverter defibrillator*, and overly formal, business-like phrasing that was not appropriate for lay audiences. Although not always technically wrong, the language used was often unnatural or difficult for everyday Gujarati speakers to understand. This ‘testing’ with PPI contributors highlighted the limitations of using digital tools alone for patient-facing materials.

**Table:** 

**Lessons for Researchers** AI translation tools may be helpful but will require validation by native speakers familiar with both medical concepts and patient communication. Lay-friendly language should be prioritised over literal accuracy when producing patient information in different languages. AI tools are better at literal accuracy than lay-friendly language.	

### Translations benefit from collaborating with PPI contributors

The experience of using ChatGPT highlighted the importance of a translation ‘validation process’[13, 35] and the essential role of PPI contributors in this process. The need for ‘validation’, however, did not end with ChatGPT, and when discussing the audio translations provided by SAHA, various issues were debated concerning the accuracy and accessibility of the translations.

For example, in the Gujarati translations, the word ‘*treadmill*’ was used, but PPI contributors felt that many Gujarati speakers may not understand this word. It was suggested that a translation for the phrase ‘*walking machine’* could be considered instead, as this would help people gain more understanding of what their participation in the study was about (undertaking an exercise or stress ECG was required during the clinical trial). PPI contributors also questioned whether terms such as *‘LifeMap’* and *‘QUEST’* should be translated, and if other words, for example, the word for ‘*vest*’ (બંડી/bandi), should not. There were concerns about whether the word ‘*bandi*’ was likely to be understood by older Gujarati speakers rather than younger people due to generational differences in the use of the language. There were other examples debated in the phrasing of the translation. For example, concerns were raised about whether a Gujarati speaker would understand the word for ‘*consent*’. Suggested other words included ‘*agree*’ (સંમત થાઓ/sammata thā’ō) or ‘*permission*’ (પરવાનગી/paravānagī).

For the Hindi translations, PPI contributors felt ‘*ves*t’ in Hindi (बनियान/baniyaan) should be used rather than English. If ‘*vest*’ in English was used, it should be followed by a description in Hindi that it was ‘*a wearable vest*’. Some content was also found to be missing from the Hindi translation, such as patients having their blood pressure taken during the exercise ECG during the study, which needed to be corrected.

The discussions of the audio translations produced by SAHA were important in validating what would be said and provided the opportunity to check that what was translated would be understood by ‘lay’ persons who spoke Gujarati and Hindi. These discussions promoted that translations were not only technically correct but also accessible to lay speakers across age groups and dialects.

**Table:** 

**Lesson for Researchers** Translation is not a one-step process; it benefits from collaborative revision involving translators and PPI contributors. Language nuances matter and should be actively explored during translations. Always check for missing content; it is easy for translators to omit important participant information.	

### Clinicians provide added value when making videos

Clinicians reviewing the audio translations identified several areas for improvement, particularly in phrasing and patient-centred clarity. For example, the Hindi translation of *‘make sure everything is okay’* (आप खुश हैं। / aap khushi hai) was flagged as vague and inaccurately phrased. Clinicians suggested replacing it with a more appropriate expression, such as (आप ठीक हैं। / Aap teekhtho hai) – *‘are you OK?’*.

Their input allowed SAHA to refine translations further before filming. The co-production process ultimately increased confidence that the final content met the expectations of PPI contributors, translators, clinicians, and the research team.

**Table:** 

**Lesson for Researchers** Clinicians play an essential role in ensuring medical accuracy and nuance in translated materials. Involving different stakeholder groups could enhance the quality and trustworthiness of the final translation materials.	

### PPI evaluation can offer insights into the quality and cultural appropriateness of the finished videos

The ratings from the PPI contributors who completed the evaluation questionnaire (*n* = 13) were positive, with ratings of four or five in most areas such as style, images, pace, information and translations (see Table 3 below). However, the headings used on the Hindi and Gujarati videos scored lower, three, suggesting improvements were needed. The overall ratings from PPI contributors were also positive, with most rating the English Video as ‘good’, and the Gujarati and Hindi videos as ‘OK’.

**Table 3:** Average PPI Ratings of the video content (1 = poor, 5 = excellent)

	Headings	Style	Images	Pace	Information	Translation
English	4	4	4	4	5	*n*/a
Hindi	3	4	4	4	4	4
Gujarati	3	4	4	4	4	4

Positive comments included that the videos were warm and authentic, that the images were good, and that the information was comprehensive. For example, one PPI contributor commented that there was

….*“good description of ECG terminology. Clear around options, i.e. male/female staff and good tone of voice and pitch. The presenter dressed in traditional outfit had a good impact”*.

Another that …

*“The sequence was great, and links to the sections of the video” and “good images were used with translation language”*.

These and other comments provided some reassurance that the videos worked well.

There were also critical comments suggesting further editing was needed. For example, *“background noise while speaking”*,* “some of the translations are not quite to the images sections”*, and the presenters’ *“eyes moving are distracting”.*

Although the final translations were seen as accurate and informative, there were still concerns from contributors that the translations were still *“too technical”*, although there was no broad agreement on this aspect. This highlights the difficulties of doing translations in different languages.

Allowing PPI contributors to evaluate the videos before the final edit proved to be essential in identifying additional changes to enhance the videos and make them more acceptable.

**Table:** 

**Lesson for Researchers** Early PPI review is valuable, but late-stage PPI evaluation is also essential for catching practical issues, e.g. sound quality, heading timings and quality. PPI contributions should extend beyond translation to include visuals, pacing, style, and presentation. Diverse views among contributors should be expected, especially disagreements about what constitutes lay-friendly language.	

## Discussion

### Translation is a collaborative process, and not just a technical task

Creating appropriate participant information videos targeted at ethnic minority groups is not without issues and is complex and multidimensional. It requires a combination of language skills, specialist medical knowledge and cultural competence. This was seen in our example of creating the LifeMap-QUEST videos, working together with PPI contributors, a community partner and clinical contributors who brought their assets to our co-production journey. This was an iterative process with each bringing complementary forms of knowledge and lived experience.

Our approach demonstrated co-production principles in practice, particularly the sharing of decisions about how the videos were produced, validated, and refined. The translation of materials is not just a technical task solved by technical means. We found, as highlighted in other studies, that digital translation tools, such as ChatGPT, do not adequately account for cultural nuances in translations [36]. Our findings also align with previous research highlighting the limitations of digital translation in health communication and the need for caution when using such tools for informed consent. For example, Panayiotou et al. (2019) found that only two translation apps were suitable for everyday conversations in healthcare settings and emphasised that such tools should not replace professional translation services [37].

### Single translations alone are insufficient

Translating written English into other languages is not simply a matter of linguistic conversion and requires interpretation and cultural competence supported by different partners. There can be an assumption that translations should be a ‘perfect match’ between languages. However, as we found, and supported by Colinas (2023), the ‘audience’ or reader of the translated materials will always bring their own ‘position’ to the reading. This will see both conscious and unconscious knowledge, values and biases’ impact on understanding [13]. When translating a written English document into another language, the challenge lies in conveying meaning rather than producing a literal, word-for-word output.

Professional translation services are seen as the ‘Gold Standard’ for providing patient information [37]. Jensen and Zethsen (2012), for example, found that professional translators (even with limited expert knowledge) tended to produce more ‘lay-friendly’ information on the drugs compared to pharmacists [38]. Our experience is that professional translation alone may be insufficient. This is backed up by other studies. Karwacka (2014), for example, identified a range of problems in the work of professional medical interpreters [39], while Brelsford et al. (2018) similarly documented challenges in translating English materials into Spanish [40]. Their findings indicate that holding a professional qualification does not necessarily prevent translators from introducing errors into research materials^5^. Other studies have shown that professionally translated written information is often produced at a readability level that exceeds what is appropriate for the target population [41].

Working with PPI contributors with language skills makes sense in this respect, not as a replacement for professional translation, such as SAHA, or expert knowledge, such as clinicians, but to complement and support the translation and interpretation of participant information in different languages.

### Co-production is a facilitator for creating culturally competent videos

Researchers may be tempted to turn to specialised services alone to undertake translations, but we would argue that, along with these services, there needs to be PPI input to get the best results. There will be different interpretations of the translated materials, and co-production helps focus on the function of the communication (for whom and what for). This will support cultural competence, considering the audience and their characteristics, e.g. age, gender, ethnicity, class, geography, education, etc. [35]. Professional translators working with several language speakers in developing and translating health materials has been suggested as a positive way to support the translation of English into a different language [40] and ensuring that sociocultural factors (which may impact interpretation and understanding), are included [42]. Our work supports this view. Co-production, in particular, the inclusion of PPI contributors is important because we believe it increases the chances that participant information in different languages will be:

- Lay-friendly rather than overly technical.
- Culturally competent.
- Accessible to different age groups and dialects.
- Reflective of local community language use.
- Sensitive to issues of trust, stigma, and historical exclusion.

### Side by side – harnessing lived experiences and different voices

There are no clear standards for producing participant information using multimedia in clinical research [18], and more studies are needed to establish best practices in this area [1]. The guidance on using languages other than English for ethical approval is also minimal, vague and underdeveloped [43], and there is a lack of clinical studies trying innovative solutions to address where English is not the first language for some potential participants [2]. This article has attempted to contribute to this conversation by describing a co-production approach undertaken by the LifeMap-QUEST study, describing the different roles in creating our videos in South Asian languages, and working side by side with our partners.

Co-production emphasises shared power and co-creation [29], which was central to our process. Partners did not simply “review” content but, working together, helped shape terminology, phrasing, examples, and visual elements of the videos. The videos included people from the same ethnic and cultural background as the target group for whom the videos were created. This was seen as a positive action that can help ensure culturally sensitive modes of communication to support rather than dissuade participants [26]. Our co-production principles also emphasise valuing different life experiences and knowledge, and operationalising this by:

- Enabling PPI contributors to challenge and refine the translations.
- Incorporating changes stemming from different lived experiences.
- Using clinician feedback to adjust terminology.
- Positioning all contributors as equal partners in the creation process.

Research teams may lack ‘cultural sensitivity’ and cultural competence or may be nervous about how to address issues of diversity [13]. Professional translators provide a necessary foundation, and clinicians ensure medical accuracy. However, neither group alone can guarantee that multi-language participant information will be comprehensible to participants. Working with PPI and community partners is therefore essential.

### Video as a tool for inclusivity

Video is seen as an underused tool in the consent process of clinical trials and provides an opportunity to address issues of inclusion, as participant-facing documents become lengthier and more complex. Using such resources to improve comprehension could help engagement and make the consent process more meaningful, particularly for those who experience literacy and language barriers to taking part in clinical studies [44]. We need to improve comprehension by making participant information more accessible and produced in different languages [13, 45].

The process of co-producing with PPI contributors, medical experts and community partners could help by increasing the cultural competence of information and helping to address underrepresentation in clinical research. Co-production provides opportunities to build capacity in research teams about the background of underrepresented groups and the languages used by these groups. Building this capacity could ensure that participant information is communicated effectively and hopefully help to establish and build trust with groups who are underrepresented in clinical trials. This is reflected in the co-production principles of reciprocity and relationship-building [29], as shared insights help shape the final outputs and we ‘learn together’ [46].

### Reflections

Completing the GRIPP2 Short Form (see Additional File 4) prompts authors to critically reflect on their work, and we would argue that this article demonstrates the importance of meaningful co-production in creating participant information videos in different languages.

#### Did our approach produce better results?

However, we recognised that final videos were not perfect and there were ongoing discussions, and occasional disagreement, about whether, despite our approach, they still reflected ‘lay’ rather than technical interpretations of the English scripts. Some writers are critical of ‘back’ translation methods and suggest other ways to provide a more rigorous assessment of translation quality [47]. For example, we didn’t use Inter-Rater Reliability (IRR) testing in our translation evaluations.

#### Was it ‘real’ co-production?

*S*ome may question the extent to which ‘real’ co-production existed in our approach, given that the videos needed to be ‘signed off’ and the research team still retain control over the final output. These are structural issues of power that exist in all research or service design and should be acknowledged. In working with PPI contributors and community partners, however, we hoped to have at least ‘enabled and empowered’ those who participated through our work and in the way we conducted our activities. This was not perfect, and there could have been additional collaboration with PPI contributors to enable them to influence more of the presentation and editing of the videos in the final stages.

#### Did we have enough funding?

A co-production approach requires time, flexibility, and adequate funding. Utilising the strengths and skills of community partners and PPI contributors to create videos requires time and resources. Our co-production journey required various meetings, paying for translators to produce different iterations of the scripts, the back and forth of getting the translations right, and then the costs of filming and editing (which all mount up). Having a PPI co-applicant and a full-time Research Associate to support this work in this respect was essential, and without these resources, co-producing the videos would not have been possible.

An important lesson, however, is to include additional costs and resources in research proposals if planning to make videos and enabling flexibility in how these funds may be used. Although we showed that it is possible to produce videos with limited resources, in hindsight, resources should have been greater. Additional back translation meetings and meetings between interpreters and the PPI Advisory Group would have been beneficial. A limitation of the work was also only having the resources to create three videos, when other South Asian languages, e.g. Urdu, would have been beneficial. Equally, other communities in Leicester, such as Polish and Somali, could have been involved in our approach.

#### Could our work be adapted?

Although our work focused on the needs of ethnic minority communities, the same principles could be adapted for other groups, such as older people with mild or moderate dementia and their carers, or children and adults with a learning disability. Future research could test our staged process of script development, accessible ‘easy read’ adaptations, forward and back translation, filming with community partners with lived experience, and involving PPI contributors in evaluating the videos. More rigorous evaluation could also be undertaken to assess which aspects of co-production best support the creation of accessible materials that could encourage wider participation in research.

## Conclusion

This article has outlined the LifeMap-QUEST study’s approach to developing participant information videos in South Asian languages. It has emphasised that such work should never be undertaken in isolation and that meaningful and culturally appropriate materials require co-production; the bringing together of the lived experiences of PPI contributors, the knowledge of community partners, and the medical understanding held by clinical experts. While participant information resources will never be perfect, we would argue that working together offers a positive way of creating participant information materials that are more likely to be accessible, culturally competent, and will support diverse communities’ inclusion in clinical research.

## Supplementary Information

Below is the link to the electronic supplementary material.

Supplementary Material 1

Supplementary Material 2

Supplementary Material 3

Supplementary Material 4

## Data Availability

No datasets were generated or analysed during the current study.

## Abbreviations

AI: Artificial Intelligence
BRC: NIHR Biomedical Research Centre
PPI: Public and Patient Involvement
ICTMC: International Clinical Trials Methodology Conference
NIHR: National Institute for Health and Care Research
QUEST: Quality assurance, User-focused, Evaluation, Safety, and Tolerability
RSS: NIHR Research Support Services
SAHA: South Asian Health Action
SCD: Sudden Cardiac Death
UK: United Kingdom
US: United States of America

## Footnotes

1 This project is funded by the National Institute for Health and Care Research (NIHR) under its Invention for Innovation (i4i) Programme (Grant Reference Number NIHR204553).

2 For more details about South Asian Health Action (SAHA) visit https://www.sahauk.org/.

3
https://le.ac.uk/research/institutes/precision-health.

4 The PPI contributors felt given the limited number of videos, these two Asian languages provided the best way of information people from the South Asian Community of the study. However, it was recognised that other Asian languages could have been considered with more resources.

5 Quality assurance in translation and interpretation is often based on a ‘qualification’ where professional services are seen as a benchmark. This article in no way wishes to suggest this is not significant or necessary but highlights how, within the context of producing clinical research participant information, involving PPI contributors and community partners will be complimentary.
