# Supplementary figures and images for: PPI in LifeMap-QUEST: an example of co-producing videos in different languages to support inclusion in a clinical study
Source: Res Involv Engagem. 2026 Mar 11;12:47. doi: 10.1186/s40900-026-00858-9 (PMC13094232; doi:10.1186/s40900-026-00858-9)

**Additional File One: ICTMC 2024**


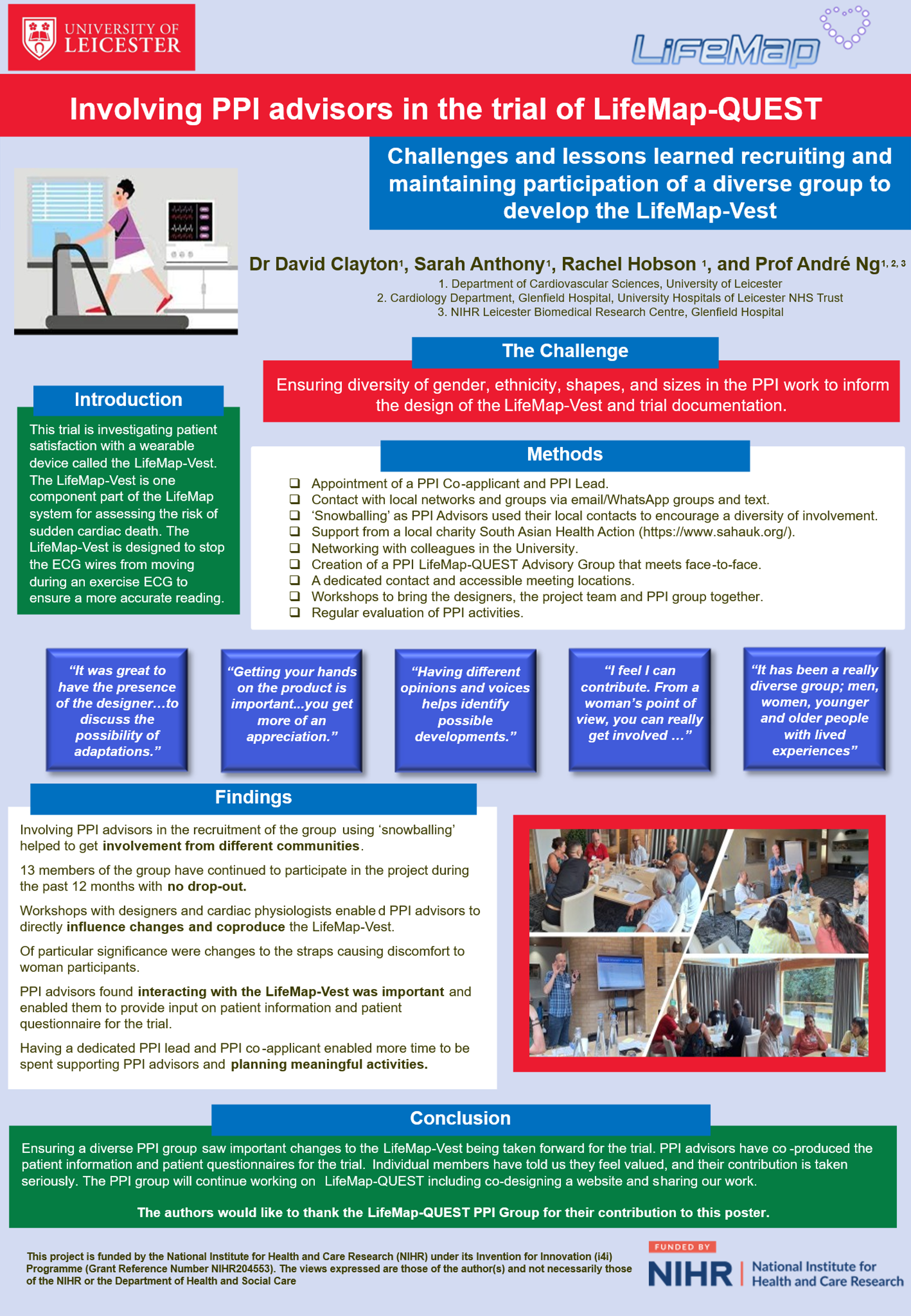

Supplement: Supplementary file 1 — Supplementary Material 1 [file 40900_2026_858_MOESM1_ESM.docx]
