# Supplementary material for: PPI in LifeMap-QUEST: an example of co-producing videos in different languages to support inclusion in a clinical study
Source: Res Involv Engagem. 2026 Mar 11;12:47. doi: 10.1186/s40900-026-00858-9 (PMC13094232; doi:10.1186/s40900-026-00858-9)
Supplement: Supplementary file 2 — Supplementary Material 2 [file 40900_2026_858_MOESM2_ESM.docx]

**Additional File Two: English Script**

| **Introduction** |
| --- |
| This video is to make you aware of the LifeMap-QUEST research study. |
| We are looking for people to take part in this study as we need their help to test a new wearable medical device called the LifeMap-Vest, which we think could benefit you and your community. |
| The vest is designed to provide a clearer and more accurate reading of the electrical activity of the heart during an exercise electrocardiogram (ECG) than readings taken with the current system. |
| The LifeMap system works by creating an electrical map of the heart to identify people with a high risk of sudden cardiac death. The LifeMap-Vest is part of the LifeMap system. |
| The LifeMap-QUEST study will aim to test whether the LifeMap-Vest is comfortable and acceptable to patients or whether changes need to be made to the design. |
| We would also like to confirm that there are no safety issues with wearing the vest before we move to the next stage of research with a much bigger study across the NHS. |
| To take part, you must be aged 18 years or above, and have been diagnosed with mild or moderate ischemic cardiomyopathy – a condition where the heart muscle has been damaged due to a lack of blood flow. |
| **What would I do in the study?** |
| You will undergo an exercise ECG whilst wearing the LifeMap-Vest and provide feedback on your experience. During an ECG, sensors are placed directly onto the skin with cables attached to look at the heart's electrical rhythm. |
| An exercise ECG is undertaken whilst walking on a treadmill. This gives doctors information about how the heart works whilst moving and can be used to detect heart rhythm problems. |
| But results from the current exercise ECG systems are sometimes not clear or as accurate, due to the cables moving around, causing interference or ‘noise’ on the ECG reading. |
| The LifeMap-Vest works by stopping the cables attached to the sensors from moving around, which reduces the electrical ‘noise’ that can interfere with the reading. |
| Wearing the LifeMap-Vest will require you to remove clothing on your chest to undergo an ECG. You can choose to be seen by female or male staff if you prefer, and you will be provided with a gown to wear if you wish. |
| **How long will the Exercise ECG take, and what does it involve?** |
| You will be invited to the hospital for a single visit. The exercise ECG will last around 10 minutes, with the whole appointment taking about 1 hour. |
| You will first be asked to read and sign a consent form. |
| You will then be asked some questions about your medical history and any medications you take. |
| A member of the research team will take information from you, such as your height, weight, chest size, date of birth and gender. |
| A cardiac physiologist will then fit you with the LifeMap-Vest and ECG sensors, and you will then start the exercise ECG. During the exercise, the ECG will be recorded whilst you walk on a treadmill. |
| There are several stages to the exercise ECG, with each lasting up to 3 minutes. To start, the treadmill will move slowly and be flat. It will speed up twice until you’re doing a brisk walk, and the tilt of the treadmill will be increased to a moderate slope. |
| Your blood pressure will be recorded at several points throughout, and you will be monitored closely during the exercise. |
| Following the exercise ECG, and once you have rested for a short while, you will be asked to complete a satisfaction questionnaire about your views on the LifeMap-Vest. |
| A member of the research team will call you 1 week after your appointment to make sure everything is okay, which should take around 10 minutes. |
| Once the study is finished, the researchers will look at the patient satisfaction questionnaires to see if patients were happy wearing the LifeMap-Vest and analyse the exercise ECG results to create the LifeMap computer diagnostic system. |
| **What do I do if I want to take part in the study?** |
| If after seeing this video you are interested in taking part in LifeMap-QUEST, speak to the research nurse who showed you this video. |
