# Supplementary material for: PPI in LifeMap-QUEST: an example of co-producing videos in different languages to support inclusion in a clinical study
Source: Res Involv Engagem. 2026 Mar 11;12:47. doi: 10.1186/s40900-026-00858-9 (PMC13094232; doi:10.1186/s40900-026-00858-9)
Supplement: Supplementary file 3 — Supplementary Material 3 [file 40900_2026_858_MOESM3_ESM.docx]

**Additional File Three: Evaluation of the videos for the LifeMap-QUEST study**

**Video rated (Please circle)**  English Hindi Gujarati

**How would you rate this video?**

**(1 being poor and 5 being great). Please tick one box only.**

|  | 1 | 2 | 3 | 4 | 5 |
| --- | --- | --- | --- | --- | --- |
| The headings |  |  |  |  |  |
| The style - ‘talking head’ |  |  |  |  |  |
| The sound quality |  |  |  |  |  |
| The images used in the videos |  |  |  |  |  |
| The places where images were used |  |  |  |  |  |
| The pace |  |  |  |  |  |
| The translation |  |  |  |  |  |
| The length |  |  |  |  |  |
| There was enough information given |  |  |  |  |  |
| The information was easy to understand |  |  |  |  |  |

**Your overall view of the video? Tick one box only.**

|  | I would rate this video as poor and do not think it will encourage people to participate in LifeMap-QUEST. I would scrap it. |
| --- | --- |
|  | The video is OK but needs more editing before it will encourage people to participate in LifeMap-QUEST. |
|  | I would rate this video as good. If I had seen this video, I would want to take part in the LifeMap-QUEST study. |
|  | I would rate this video as fantastic and could watch it again and again. Can I have a copy? |
|  | Any comments to help us improve the videos (please continue overleaf if needed) |
