# Supplementary material for: PPI in LifeMap-QUEST: an example of co-producing videos in different languages to support inclusion in a clinical study
Source: Res Involv Engagem. 2026 Mar 11;12:47. doi: 10.1186/s40900-026-00858-9 (PMC13094232; doi:10.1186/s40900-026-00858-9)
Supplement: Supplementary file 4 — Supplementary Material 4 [file 40900_2026_858_MOESM4_ESM.docx]

**Additional File 4: GRIPP2 Short Form**

| **Section and Topic** | **Item** |
| --- | --- |
| **1: Aim**  Report the aim of PPI in the study | LifeMap-QUEST is a project designed to test a new medical device (the LifeMap-Vest) for patient satisfaction and ECG signal clarity. This vest is part of a broader system aimed at diagnosing individuals at risk of sudden cardiac death. Worn during an exercise ECG, the vest stabilises wires to improve signal accuracy. PPI was essential for co-designing the vest and refining patient-facing materials for the clinical study, including the multilingual videos described in this article. Recognising that written English materials can be inaccessible for many potential participants, particularly those with limited literacy or for whom English is not a first language, the research team worked collaboratively with the LifeMap-QUEST PPI Advisory Group, a local community charity, and clinicians to develop “talking‑head” videos in English, Gujarati, and Hindi for the South Asian Community. Guided by principles of co-production, the approach sought to ensure the materials were accurate, relevant, and culturally meaningful. This article describes the co-production process, the role and contributions of partners, and some lessons for developing inclusive participant‑information video resources; it does not evaluate the videos themselves but offers the work as an example of good involvement practice in clinical trials. |
| **2: Methods**  Provide a clear description of the methods used for PPI in the study | A full-time PPI Lead was appointed, and a PPI Advisory Group was set up for the LifeMap-QUEST study. Recruitment to the group was conducted via local groups and community contacts. PPI contributors were encouraged to recruit others through a snowballing approach. The group has a high number of people from the South Asian Community. The group attended monthly in-person meetings and practical workshops to try on the vest and co-develop patient materials, including creating videos. Creating the videos used a two-stage process: first, a participatory translation stage, and second, a filming and evaluation stage. Participatory translation involved PPI contributors, community partners, clinicians and the research team working together through meetings and email discussions to use forward/back translations into Gujarati and Hindi. Contributors reviewed the accuracy, cultural appropriateness, and clarity of the translations. In the second stage, South Asian Health Action and the University of Leicester’s Institute for Precision Health collaborated on planning, filming and editing the “talking‑head” videos. PPI contributors then viewed and discussed the videos, completing brief questionnaires to guide refinements for the editors. A final review meeting ensured the correct placement of headings and images before the videos were signed off for use in the LifeMap‑QUEST clinical study. |
| **3: Study results Outcomes**  Report the results of PPI in the study, including both  positive and negative outcomes | The co-production process led to improvements in the translation accuracy, cultural appropriateness, and clarity of the videos, with PPI contributors playing a central role in identifying limitations of early translation. In particular, contributors highlighted important linguistic nuances, such as unclear terms prompting more accessible alternatives, while clinicians added value by correcting ambiguous phrasing. PPI evaluation of the draft videos generated largely positive feedback, with high ratings for style, images, pace, and information, and comments noting warmth, authenticity, and cultural relevance. However, lower scores existed for headings, and concerns were raised about background noise and the mismatched timing of visuals and translations. There was some disagreement about whether the language remained “too technical”, highlighting the challenges of translating medical concepts across languages. Overall, PPI involvement helped strengthen the quality of the videos, ensured important corrections before final editing, and revealed both the benefits and complexities of multilingual co-production. |
| **4: Discussion and conclusions**  Outcomes—Comment on the extent to which PPI influenced the study overall. Describe positive and negative effects. | PPI was highly influential in LifeMap-QUEST. Collaboration among partners led to the creation of videos tailored to the South Asian community, which would not have been produced without PPI input. Contributors helped identify significant limitations in digital and professional translations, ensuring the final materials were more lay-friendly, culturally competent, and reflective of local language use across ages and dialects. Their involvement was based on core co-production principles, enabling shared decision‑making, challenging terminology, refining scripts, and ensuring that visuals and examples resonated with the South Asian communities the study aimed to reach. The effectiveness of multilingual videos in supporting underrepresented groups in clinical trials remains to be evaluated. Nonetheless, the co-production process was positive and contributed to broader discussions on inclusive PPI in health research. The study underscored the complexity of translating patient information and the importance of involving PPI contributors, clinicians and community partners rather than just relying on professional translation services. |
| **5: Reflections/critical perspective**  Comment critically on the study, reflecting on the things that went well  and those that did not, so others can learn from this experience | The co-production approach is assumed to have strengthened the accuracy, cultural relevance, and accessibility of the LifeMap‑QUEST videos. However, the process revealed challenges, including disagreements about whether translations were truly “lay-friendly,” the limitations of back‑translation methods, and structural power imbalances given that the research team retained final sign-off. Resource constraints restricted the number of languages produced and limited the opportunity for additional translation checks, highlighting the need for more funding, time, and flexibility to support genuine co-production. Despite these limitations, the principles and staged approach used here could be adapted for other underserved groups, and future work should test these methods more rigorously to understand how co-production can support the creation of accessible, inclusive research materials. |
